# Intratumoral B cell and interferon signatures in newly diagnosed glioblastoma are associated with longer survival in patients treated with SurVaxM

**DOI:** 10.1007/s00262-025-04193-y

**Published:** 2025-10-09

**Authors:** Henry G. Withers, Sheila A. Figel, Jingxin Qiu, Manmeet Singh Ahluwalia, David Reardon, Ajay P. Abad, William T. Curry, Eric T. Wong, David M. Peereboom, Andrew Dhawan, Song Liu, Michael J. Ciesielski, Robert A. Fenstermaker

**Affiliations:** 1 Department of Biostatistics and Bioinformatics, Roswell Park Comprehensive Cancer Center, Buffalo, NY USA; 2 Department of Neurosurgery, Roswell Park Comprehensive Cancer Center, Elm and Carlton Streets, Buffalo, NY 14263 USA; 3 MimiVax, Inc, Buffalo, NY USA; 4 Department of Pathology, Roswell Park Comprehensive Cancer Center, Buffalo, NY USA; 5 Miami Cancer Institute, Baptist Health South Florida, Miami, FL USA; 6 Department of Medical Oncology, Dana-Farber Cancer Institute, Boston, MA USA; 7 Department of Neuro-Oncology, Roswell Park Comprehensive Cancer Center, Buffalo, NY USA; 8 Massachusetts General Hospital Cancer Center, Harvard Medical School, Boston, MA USA; 9 Department of Neurology, Beth Israel Deaconess Medical Center, Boston, MA USA; 10 Rose Ella Burkhardt Brain Tumor Center, Cleveland Clinic, Cleveland, OH USA; 11 Neurological Institute, Cleveland Clinic, Cleveland, OH USA; 12 Present Address: Brown University Health Cancer Center, Providence, RI USA

**Keywords:** Glioblastoma, Immunotherapy, Survivin, Gene Expression Profiling, Transcriptome, Exome, Vaccines, Interferons, B-Lymphocytes, SurVaxM

## Abstract

Glioblastoma (GBM) has proved difficult to treat, and there is dire need for more effective therapies. In a single arm phase IIa trial (NCT02455557), treatment of newly diagnosed GBM patients with the peptide vaccine SurVaxM resulted in promising median progression-free and overall survival. To investigate molecular features that associate with GBM responsiveness to SurVaxM, retrospective whole exome and RNA sequencing was performed on patient tumors (n = 34) collected prior to standard of care treatment plus SurVaxM. Differential gene expression and mutational profiles were characterized between patients with short-term (OS < 18 months) or long-term (OS ≥ 18 months) overall survival. Greater expression of interferon, complement, and humoral immunity signatures were associated with long-term survival. Deconvolution of transcriptomes identified enrichment of intratumoral memory B cell populations in long-term survivors that were validated by CD20 staining in matched samples. A five-gene expression signature and a B cell specific signature predicted survival within the SurVaxM-treated cohort, however, these signatures were not associated with improved outcomes in a similarly treated population obtained from The Cancer Genome Atlas (TCGA) that did not receive immunotherapeutic intervention. Although prospective validation is ongoing, the findings in this discovery cohort specify molecular features of GBM associated with better overall survival and potential responsiveness to immunotherapy with SurVaxM.

**Supplementary Information:**

The online version contains supplementary material available at 10.1007/s00262-025-04193-y.

## Introduction

Glioblastoma (GBM) is the most common primary brain malignancy representing 14.2% of all brain tumors. Fewer than 7% of GBM patients survive 5 years following diagnosis [1]. Standard of care (SOC), involving surgical resection, chemoradiation, and adjuvant temozolomide (TMZ) was associated with a median survival time of 15.8 months [2]. Glioblastomas have been subjected to extensive molecular analyses revealing robust genetic and transcriptomic alterations that classify isocitrate dehydrogenase (IDH) wild-type tumors into three presently recognized distinct subtypes: classical, mesenchymal and proneural [3–5]. Although the molecular signatures associated with these subtypes are highly consistent, the subtypes are not associated with distinct survival outcomes and do not direct therapeutic interventions [4, 5]. A small number of key features have reliably been found to influence clinical outcomes. These include mutation of the isocitrate dehydrogenase gene (*IDH1*) and promoter methylation of the *MGMT* (O6-methylguanine-DNA methyltransferase) DNA repair gene, which is associated with an enhanced response to the alkylating agent TMZ [6, 7].

Recently, the peptide-conjugate vaccine SurVaxM, which targets the survivin (*BIRC5*) tumor antigen, has shown promise in the treatment of newly diagnosed glioblastoma (nGBM). In a multi-institutional phase IIa clinical trial (NCT02455557), nGBM patients underwent gross total or near-total resection of contrast-enhancing tumor and chemoradiation followed by treatment with SurVaxM plus adjuvant TMZ. Of 63 patients treated in this single-arm study, median progression-free survival (mPFS) was 11.4 months and median overall survival (mOS) was 25.9 months as measured from the time of first post-chemoradiation vaccination [8]. To assess whether tumor-specific molecular signatures contribute to improved outcomes in SurVaxM-treated nGBM patients, we performed retrospective genomic and transcriptomic assessments of pre-treatment tumors from 34 patients enrolled in the study who were either short-term (OS < 18 mo) or long-term (OS ≥ 18 mo) survivors. The initial findings described herein define a pre-existing molecular state in nGBM that is potentially primed for immune response upon chemoradiation and SurVaxM vaccination leading to extended OS. Key gene expression signatures point towards active interferon signaling, extracellular matrix remodeling, glycan modulation, and enhanced humoral and complement immune system components in tumors from long-term survivors. Furthermore, tumor infiltration of B cells correlated with improved outcomes for these patients. These findings justify future analyses in a larger cohort of patients from a randomized, placebo-controlled trial of SurVaxM.

## Materials and methods

### Clinical trial

Data and samples were obtained from nGBM patients treated in a phase IIa clinical trial (NCT02455557) with surgery, chemoradiation, and SurVaxM plus TMZ as described in [8]. The study adheres to the Declaration of Helsinki and received approval from the institutional review boards at each of the participating hospitals: Beth Israel Deaconess Medical Center, Cleveland Clinic, Dana-Farber Cancer Institute, Massachusetts General Hospital, and Roswell Park Comprehensive Cancer Center. All participants signed an informed consent prior to participation in the study.

### Immunohistochemistry

Tissue slides from 23 patients in the SurVaxM trial were available for immunohistochemical analysis. Slides were added to Leica Bond Rx, deparaffinized with Bond Dewax Solution (Leica AR9222), rinsed in water, and exposed to Bond Epitope Retrieval 2 (Leica AR9640). Slides were blocked using peroxide block from Bond Polymer Refine Detection kit (Leica DS9800, RRID:AB_2891238) and then incubated with CD20 primary antibody (Agilent M0755, RRID:AB_2282030) for 20 min at 1/1000 dilution. Post Primary (Leica DS9800, RRID:AB_2891238) was applied followed by Polymer for 8 min each, diaminobenzidine for 10 min for visualization, counterstained with Hematoxylin for 8 min, and placed into water. Slides were dehydrated, cleared, coverslipped, and imaged at 40 × magnification using an Aperio AT2 slide scanner (RRID:SCR_021256). Entire slide images (one slide per patient) were assessed for CD20 staining patterns described as negative, rare positive staining, or focal positive staining by a pathologist (J. Qiu) blinded to the overall survival status and B cell transcriptomic signature score of each sample.

### Whole exome analysis

Formalin fixed and paraffin embedded (FFPE) tumor samples (n = 34) were submitted to Tempus (Chicago, IL) for whole exome sequencing using the xE pipeline to identify somatic mutations and copy number alterations as described in Beaubier et al. (2019) [9]. In the absence of a matched normal sample, variants called by Freebayes (RRID:SCR_010761) and Pindel (RRID:SCR_000560) are classified as germline or somatic based on a Bayesian model of the likelihood that the variant is somatic, following an approach similar to methods described by Halperin et al. (2017) and Sun et al. (2018) [10, 11]. Uncertain variants are subsequently treated as somatic variants for filtering purposes. Only alterations with a variant allele frequency ≥ 10% are reported.

### RNA-seq analysis

Tumor samples (n = 33) isolated from patients during craniotomy were formalin fixed/paraffin embedded (FFPE). RNA extraction, library preparation, and paired-end bulk RNA sequencing of samples was performed by Tempus (Chicago, IL) as described in Beaubier et al. (2019) [9]. Reads were aligned to reference genome GRCh38 (GENCODE v38, RRID:SCR_014966) with Salmon v1.1.0 (RRID:SCR_017036) [12]. Transcript counts were consolidated and annotated using the Bioconductor package Tximeta [13]. LT versus ST differentially expressed genes were modeled with DESeq2 (RRID:SCR_015687) while accounting for age, sex, *MGMT* methylation status, and molecular subtype as covariates [14]. Classical, mesenchymal, and proneural GBM signatures were assigned to IDH1 wild type samples based on the greatest positive Gene Set Variation Analysis (GSVA) enrichment score [5, 15]. Gene set enrichment analysis (GSEA) was performed using the fgsea BioConductor R package (RRID:SCR_020938) applied to hallmark (H), curated (C2), and ontology (C5) gene sets available in the Molecular Signatures Database (MSigDB, RRID:SCR_016863) [16–18].

### Immune deconvolution

The transcript per million (TPM) data for all RNA-sequencing samples were submitted to the TIMER2.0 webtool (http://timer.cistrome.org/; RRID:SCR_018737) for immune deconvolution [19]. A comprehensive summary of deconvolution methods employed by TIMER2.0 is available from Strum et al., 2019 [20].

### TCGA GBM dataset

The Cancer Genome Atlas (TCGA, RRID:SCR_003193) gene expression data and clinical data for GBM tumors were retrieved from National Cancer Institute (NCI) Genomic Data Commons (GDC, RRID:SCR_014514) using the TCGAbiolinks Bioconductor R package (RRID:SCR_017683) [21–24]. Specifically, the harmonized data for gene expression quantification from the “STAR—Counts” standardized workflow were collected for unique primary tumor samples with overall survival data available (n = 154).

### Statistical analysis

All statistical analyses and visualizations were completed within the R software environment (RRID:SCR_001905), and reporting of statistical tests and associated results are provided in the relevant text and figure captions. A *p*-value threshold of < 0.05 was considered significant for all statistical tests. Differentially expressed genes with an absolute log_2_ fold change > 1.0 and false discovery rate < 0.1 (Benjamini–Hochberg corrected *p*-value) were identified as significant. All gene sets reported for GSEA were significant with a Benjamini–Hochberg adjusted *p*-value < 0.05. Age, *MGMT* methylation status, and B cell signature score were significantly associated with OS by univariate analysis and were included as covariates in a multivariate Cox proportional hazards model of OS. The model is stratified on *MGMT* methylation status because this covariate violated the proportionality assumption with a significant Schoenfeld residual test (*p* = 0.042) and by inspection of the log-minus-log plot.

## Results

### Molecular profiling of a representative patient cohort

**Fig. 1:**
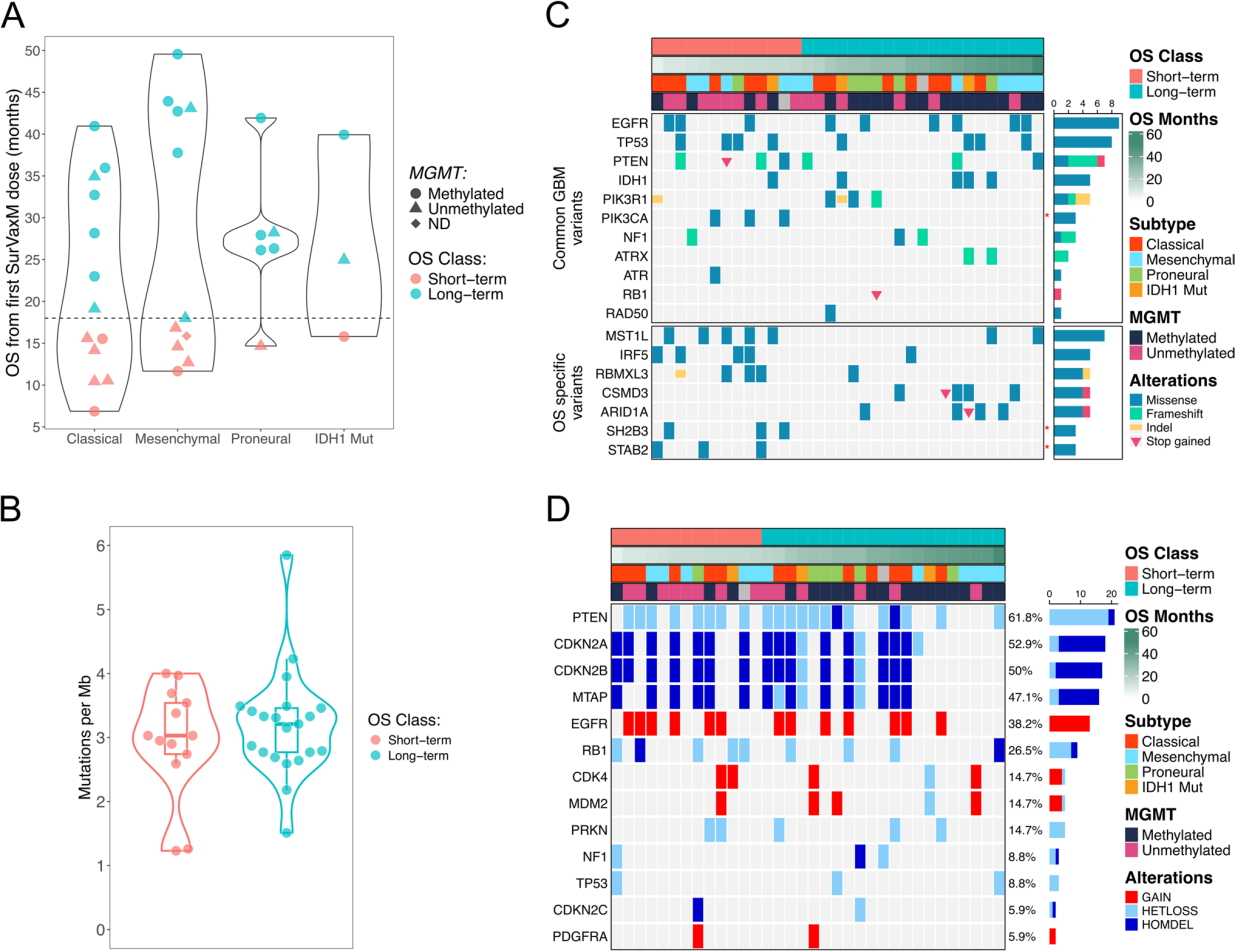
*Landscape of genetic alterations in GBM tumors prior to treatment* **A** Violin plot of OS from initial SurVaxM dose in months across all molecular subtypes [5] with OS class (ST = red, LT = blue) and MGMT methylation status denoted (point = methylated, triangle = unmethylated, and diamond = not determined). **B** Violin plot of tumor mutational burden between OS classes as measured by mutations per megabase of genomic sequence (ST = red, LT = blue). Differences between OS classes are not significant by student’s t-test (n = 34, *p* = 0.492). **C** Oncoprint of nonsynonymous somatic mutations for genes commonly altered in GBM or variants specifically enriched in either LT (OS ≥ 18 mo) or ST (OS < 18 mo) samples and present in at least 3 samples. Annotations denote OS class, OS in months from initial SurVaxM dose, assigned molecular subtype [5], and methylation status of *MGMT*. Row-wise frequency of gene alterations are indicated by horizontal bar plot. Red asterisks indicate significance between OS classes by Fisher’s exact test (*p* < 0.05, n_ST_ = 13, n_LT_ = 21). **D** Oncoprint of copy number alterations for genes commonly altered in GBM (HETLOSS = heterozygous loss; HOMDEL = homozygous deletion). Annotations denote OS class, OS in months from initial SurVaxM dose, assigned molecular subtype [5], and methylation status of *MGMT*. Row-wise frequency of gene alterations are indicated by percentages and horizontal bar plot

In a phase IIa clinical trial (ClinicalTrials.gov: NCT02455557), nGBM patients were treated with SOC (tumor resection, chemoradiation, and adjuvant temozolomide) followed by a vaccine priming phase of four subcutaneous doses of SurVaxM in Montanide ISA-51 administered once every two weeks [8]. Subsequent maintenance doses of SurVaxM were administered every 3 months concurrently with adjuvant TMZ until tumor progression. For 63 enrolled patients, a subset of tissue samples from craniotomy prior to treatment were available for genomic (n = 34) and transcriptomic (n = 33) analyses. These samples included 11 females (32%) and 23 males (68%), ranging in age from 24 to 76 years (Table 1). Three tumors (9.1%) harbored *IDH1* mutations (R132H) leading to their categorization as grade 4 astrocytoma, IDH-1 mutant in the most current WHO classification scheme. Gene set variation analysis (GSVA) of tumor transcriptional profiles identified samples as classical (n = 13, 39%), mesenchymal (n = 11, 33%), or proneural (n = 6, 18%) subtypes (Table 1) [5, 8, 15]. The mOS for all SurVaxM-treated patients in this trial was 25.9 months, and the mOS for the subset of molecularly profiled patients was 25.6 months [8]. The profiled patients were classified as short-term (ST; OS < 18 mo; n = 13) or long-term (LT; OS ≥ 18 mo; n = 21) survivors by maximally selected log-rank statistic cutpoint analysis (Table 1 and Supplementary Fig. 1A). Genomic and transcriptional data were compared between these two OS groups to identify underlying factors contributing to increased survival. Both age and *MGMT* methylation status correlated significantly with OS consistent with results reported in the whole study cohort data [8]. For the 21 LT patients, 15 (71%) tumors exhibited me-*MGMT*; whereas, me-*MGMT* was detected in only 4 (33%) of 13 ST patient tumors (Table 1). The OS of patients did not differ between molecular subtypes; however, the OS of patients with mesenchymal tumors is distinctly bimodal and largely determined by *MGMT* methylation status (Fig. 1A and Supplementary Fig. 1B).

**Table 1:** Patient characteristics stratified by long-term and short-term overall survival status

Survivor Status	Overall, N = 34^1^	Short-term, N = 13^1^	Long-term, N = 21^1^	*p*-value^2^
Age	60 (24, 76)	67 (27, 76)	54 (24, 69)	0.010
*Sex*				0.7
Female	11 (32%)	5 (38%)	6 (29%)	
Male	23 (68%)	8 (62%)	15 (71%)	
*Molecular Subtype*				0.7
Classical	13 (39%)	6 (46%)	7 (35%)	
Mesenchymal	11 (33%)	5 (38%)	6 (30%)	
Proneural	6 (18%)	1 (7.7%)	5 (25%)	
IDH1 Mut	3 (9.1%)	1 (7.7%)	2 (10%)	
Unknown	1	0	1	
*IDH1 Status*				> 0.9
Mutant	3 (8.8%)	1 (7.7%)	2 (9.5%)	
Wild Type	31 (91%)	12 (92%)	19 (90%)	
*MGMT Status*				0.033
Methylated	19 (58%)	4 (33%)	15 (71%)	
Unmethylated	14 (42%)	8 (67%)	6 (29%)	
Unknown	1	1	0	
*Survivin IHC %*				0.2
1–4	2 (5.9%)	0 (0%)	2 (9.5%)	
5–9	10 (29%)	2 (15%)	8 (38%)	
10–19	16 (47%)	7 (54%)	9 (43%)	
≥ 20	6 (18%)	4 (31%)	2 (9.5%)	
SurVaxM doses	7.5 (2.0, 18.0)	5.0 (2.0, 8.0)	11.0 (4.0, 18.0)	< 0.001
*Initial KPS score*				0.8
70	1 (2.9%)	0 (0%)	1 (4.8%)	
80	14 (41%)	5 (38%)	9 (43%)	
90	16 (47%)	6 (46%)	10 (48%)	
100	3 (8.8%)	2 (15%)	1 (4.8%)	

^1^Median (Range); n (%)

^2^Wilcoxon rank sum test; Fisher’s exact test; Pearson’s Chi-squared test

### Baseline genetic alterations defined between overall survival classes

Tumors were subjected to whole exome sequencing to identify genomic alterations unique to ST or LT survival classes. There was no significant difference in tumor mutational burden between ST and LT cohorts (two-sided Student’s t-test, n_ST_ = 13, n_LT_ = 21, *p* = 0.492; Fig. 1B). Nonsynonymous somatic alterations were observed for genes commonly altered in GBM with the highest mutation rates detected for *EGFR* (26.5%), *TP53* (23.5%), and *PTEN* (20.6%) (Fig. 1C). Among these common GBM alterations, mutations in *PIK3CA* (8.8%) were significantly associated with ST samples (Fisher’s exact test, *p* = 0.0478). An unbiased analysis of all differential alterations between LT and ST samples specifically identified *SH2B3* and *STAB2* missense mutations as significantly associated with ST tumors (Fisher’s exact test, *p* = 0.0478). The lymphocyte adaptor protein (LNK encoded by *SH2B3*) is a negative regulator of JAK-STAT signaling through direct interactions with JAK2 and JAK3, but LNK has also been implicated as an adaptor protein mediating STAT3 signaling from IL-6/gp130 in GBM [25, 26]. Stabilin-2 (*STAB2*) is the primary clearance receptor controlling endocytic turnover of hyaluronan (HA), a key glycosaminoglycan of ECM that is associated with cell proliferation, invasion, angiogenesis, lymphocyte trafficking, and immune protection in GBM [27, 28]. Although not significant due to sample size limitations, several additional genes were substantially enriched for mutations in either LT (*ARID1A* and *CSMD3*) or ST (*MST1L*, *IRF5*, and *RBMXL3*) tumors. Prominent among these alterations is a single variant of uncertain significance (rs113806178; R191Q) found in all cases of altered interferon regulatory factor 5 (*IRF5)* present in 4 ST samples (38%) and 1 LT sample (4.8%). Conversely, LT tumors frequently harbored missense mutations in *ARID1A* (23.8%), a member of the BAF complex known to interact with EZH2. *ARID1A* deficiencies are associated with increased microsatellite instability, higher mutational burden, greater immune infiltration, and improved response to immune checkpoint blockade in other cancer types [29, 30]. Tumors from LT survivors also contained missense and gain of stop codons in the complement control gene *CSMD3* (23.8% of LT samples). In mice, the loss of *Csmd3* results in elevated expression of complement proteins in the cortex along with genes associated with angiogenesis, inflammatory reactions, and cell cycle in microglia, endothelial cells, and astrocytes [31].

Copy number alterations (CNAs) were present in all 34 GBMs. Although not differential between LT and ST classes, chromosomal alterations commonly observed in GBMs were present across the cohort, including high frequency loss of chromosomes 9p (53.8% ST; 42.8% LT), 10 (61.5% ST; 52.4% LT), 13 (38.5% ST; 19% LT), and 22 (30.8% ST; 23.8% LT; Supplementary Fig. 2A). Gene-specific copy number alterations commonly found in GBM were detected across OS classes, including *PTEN* loss (61.8%), *EGFR* gain (38.2%), and homozygous deletion in 9p21.3 containing *CDKN2A*, *CDKN2B*, and *MTAP* (Fig. 1D). There were no significantly different CNAs observed between OS cohorts (Fisher’s exact test; ST = 13, LT = 21, p > 0.05); however, several genes and loci were moderately enriched for CNAs in LT or ST samples (Supplementary Fig. 2A and B). Although widespread alterations of chromosomes 10 and 13 were reported across many of the tumors, a collection of genes on chromosome 10 (*AGAP9*, *FAM25C*, *SYT15*, *GPRIN2*, and *NPY4R*) and *TBC1D4* on chromosome 13 exhibited loss of heterozygosity more frequently in ST tumors. Heterozygous loss of a chromosome 21q locus containing *TSPEAR* and paralogs for a subfamily of keratin associated proteins (*KRTAP10*) was unique to a subset of LT samples (14.7%). Similarly, another keratin associated protein, *KRTAP9-3*, was lost from chromosome 17 at high frequency in LT tumors (33.3% LT and 7.8% ST). Other genes predominantly lost in LT samples were *MUC4*, *TRIM49C*, *PSG3*, *PSG8*, and *ZNF595*.

### Gene expression profiling of pre-treatment tumors from long-term and short-term survivors treated with SurVaxM

**Fig. 2:**
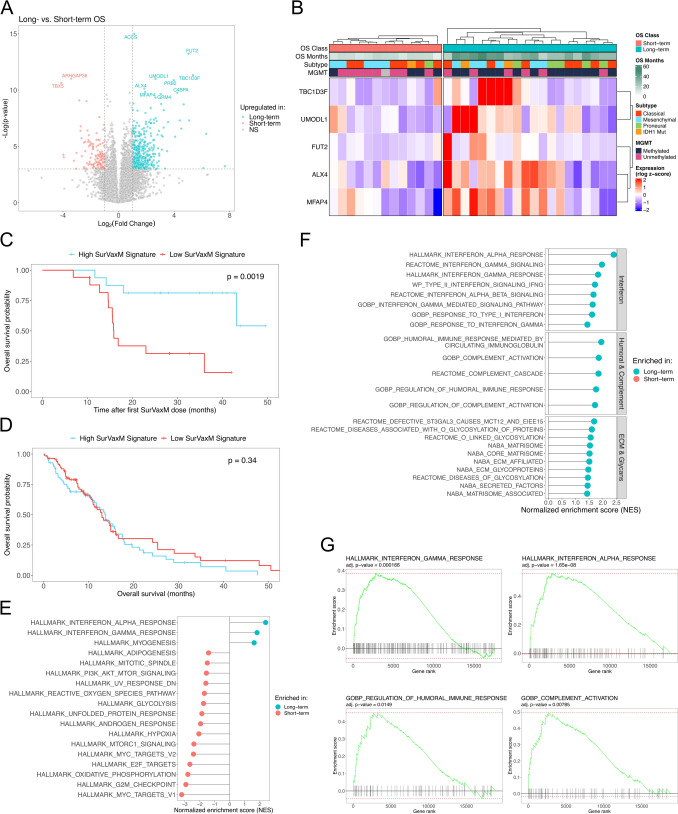
*RNA-seq profiling of long-term vs short-term SurVaxM survivors* **A** Volcano plot of differential gene expression between LT (OS ≥ 18 mo; blue) and ST (OS < 18 mo; red) tumors from RNA-seq accounting for age, sex, *MGMT* methylation status, and molecular subtype [5] as modeling covariates. Dashed lines represent thresholds for *p*-value < 0.05 and |log_2_ fold change|> 1.0. Gene symbol labeled points identify differentially expressed genes with FDR < 10% (Benjamini–Hochberg corrected). **B** Heatmap of row-scaled rlog normalized expression values for top selected differentially expressed genes that stratify patients by OS class. Annotations denote OS class, OS in months from initial SurVaxM dose, assigned molecular subtype [5], and methylation status of *MGMT*. Kaplan Meier analysis of OS for the SurVaxM cohort (**C**; n = 34) or TCGA GBM dataset (**D**; n = 154) stratified by positive (high SurVaxM signature; blue) or negative (low SurVaxM signature; red) gene set variation analysis score of 5-gene differential expression signature. Respective p-values reported for log rank statistic. Gene set enrichment analysis reporting significantly enriched HALLMARK (**E**) or selected category C2 and C5 (**F**) gene sets from functionally similar groups (interferon, humoral/complement, or extracellular matrix/glycans) from the Molecular Signatures Database (MSigDB) in LT (blue) or ST (red) tumors. **G** Gene set enrichment plots with cumulative enrichment scores across comparative gene ranks (LT vs ST) for gene sets related to interferon gamma, interferon alpha, humoral immune response, and complement activation. Adjusted p-values are reported for respective gene sets (Benjamini–Hochberg corrected)

To examine whether specific molecular determinants underlie more favorable outcomes upon SurVaxM treatment, expression profiling of patient tumors (n = 33) was performed. Transcriptomes were compared between LT (n = 20) and ST (n = 13) groups for differential gene expression while accounting for age, sex, *MGMT* methylation status, and molecular subtype as covariates (Table 1**, **Fig. 2, and Supplementary Fig. 3A). Comparative analysis of global transcriptional profiles identified broad differences in gene expression (464 upregulated and 153 downregulated genes in LT compared to ST; *p*-value < 0.05, |log_2_ fold change|> 1.0; Supplementary Table 1). Ultimately, nine genes were found to be significantly upregulated and two genes significantly downregulated in LT survivors compared with ST survivors after multiple testing correction (FDR < 10%) (Fig. 2A and Supplementary Fig. 3B). Many of the identified differentially expressed genes are understudied in glioblastoma; however, several are key regulators of tissue remodeling (*MFAP4* and *ALX4*), mesoderm development (*TBX5* and *ALX4*), and immune modulation (*FUT2*, *C4BPA*, *MFAP4*, and *GRM4*). Expression of five of these genes (*TBC1D3F, UMODL1, FUT2, MFAP4,* and *ALX4*) generally stratified patients by OS class upon unsupervised clustering (Fig. 2B). The five gene signature was scored in each sample by GSVA and OS was assessed by Kaplan–Meier analysis between low (negative) and high (positive) SurVaxM signature scores. High expression of this SurVaxM signature was significantly associated with improved OS in the SurVaxM-treated cohort (median OS high = undetermined; median OS low = 15.9 months; log rank test, *p* = 0.0019; Fig. 2C). Comparatively, the SurVaxM signature score assessed in 154 primary GBM tumors publicly available in the TCGA database did not stratify patients by OS (mOS = 13.3 months; Fig. 2D) [24]. The TCGA cohort were similarly treated with radiation, TMZ, or a combination thereof but lacked immune targeted interventions. Although limited by sample size, these data suggest an existing tumor state primed to respond favorably to immune modulation specifically induced by SurVaxM vaccination in a subset of patients.

### Adaptive and innate immune-related pathways are enriched in tumors from long-term survivors

Further investigation of underlying expression profiles by gene set enrichment analysis (GSEA) revealed distinct enrichment of HALLMARK gene signatures related to immune response in samples obtained from LT survivors, including gene sets for interferon-gamma (IFN-γ) and interferon-alpha (IFN-α) response in addition to myogenesis (Fig. 2E). There is notable overlap between the leading-edge genes of IFN-γ and IFN-α response signatures (overlap n = 30) that include genes associated with immune infiltration, activation, and antitumor response, including *CXCL9, CXCL10, CXCL11, IRF7, IRF9, STAT1, STAT2, JAK2, TRIM25, TRIM26,* and *LY6E*. Amongst the gene sets upregulated in ST patient tumors were several signaling and regulatory pathways involving tumorigenicity, proliferation, and aggressiveness, including Myc and E2F transcriptional targets, G2M checkpoint, mitotic spindle formation, and PI3K/AKT/MTOR signaling axis. Additionally, the ST samples were enriched for HALLMARK gene sets representing metabolic pathways known to drive oncogenesis, including hypoxia, glycolysis, and reactive oxygen species. Expansion of GSEA to include curated and gene ontology gene sets resulted in enrichment of LT tumors for multiple gene sets from functionally related categories that impact antitumor immunity (Fig. 2F and G). Consistent with HALLMARK GSEA results, six additional IFN-γ and IFN-α gene sets from different source databases (Reactome and GOBP) were enriched in LT tumors. The LT transcriptional profiles were significantly enriched in pathways related to the extracellular matrix (ECM) and glycosylation of proteins, both critical in regulating immune cell trafficking, differentiation, and activation [32]. Notably, the top expressed genes in LT tumors include an ECM protein, microfibrillar-associated protein 4 (*MFAP4*), involved in cell adhesion and intracellular interactions, and fucosyltransferase 2 (*FUT2*) a critical modifier of glycans on selectins and other ECM associated proteins responsible for leukocyte adhesion, extravasation, macrophage polarization, B cell development, and antibody-dependent cellular cytotoxicity [33–37]. The third functional category of pathways highly enriched in LT samples includes gene sets for humoral immune response and the complement system, both potentially integral pathways to SurVaxM’s mechanism of action. Furthermore, complement component 4 binding protein alpha (*C4BPA*) is significantly upregulated in LT tumors, and *C4BPA* is an established binding partner of CD40 on antigen presenting B cells, macrophages, and dendritic cells ultimately leading to B cell class switching, germinal center formation, and CD4^+^/CD8^+^ T cell proliferation (Fig. 1A) [38–40].

### Immune deconvolution indicates higher B and T cell proportions in treatment-naïve tumors of SurVaxM long-term survivors

**Fig. 3:**
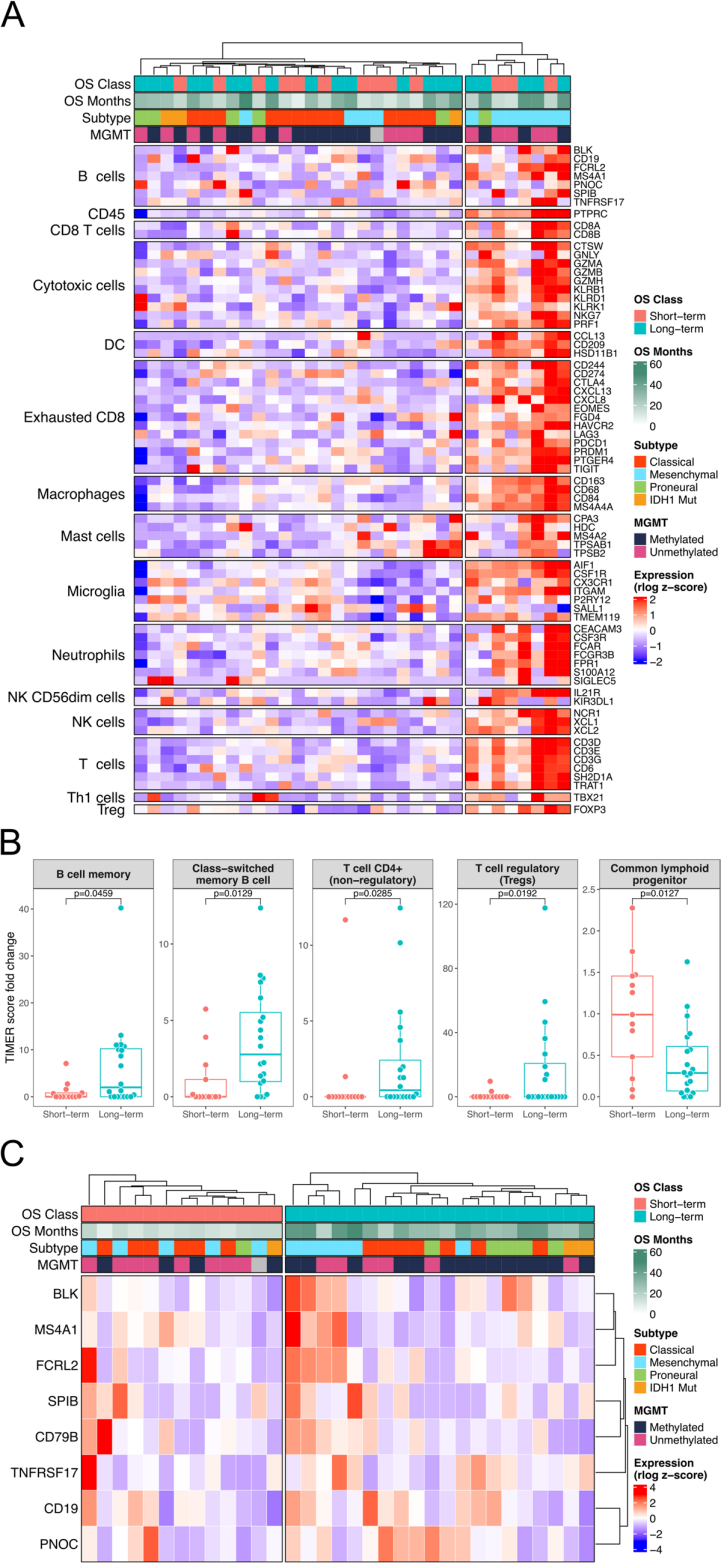
*Immune deconvolution analysis of transcriptomes identifies B cell signature in GBM tumors of LT survivors* **A** Heatmap of row-scaled rlog normalized expression values for immune compartment signatures defined by Danaher et al. (2017) [41] plus a microglia signature. Samples are delineated as overall high or low immune infiltration expression signal by splitting on the first dendrogram level after hierarchical clustering by Euclidean distance. Annotations denote OS class, OS in months from initial SurVaxM dose, assigned molecular subtype [5], and methylation status of *MGMT*. **B** Normalized TIMER2.0 immune deconvolution signatures with significant differences between LT and ST OS classes (Wilcoxon rank-sum test, n_ST_ = 13, n_LT_ = 20, p < 0.05). Boxplots represent the median and interquartile range. **C** Heatmap of row-scaled rlog normalized expression values for an 8 gene modified B cell signature derived from Danaher et al. (2017) and Budczies et al. (2021) [41, 43]. Annotations denote OS class, OS in months from initial SurVaxM dose, assigned molecular subtype [5], and methylation status of *MGMT*

The tumor microenvironment composition of patient tumors was explored further given the immunomodulatory role of SurVaxM and the prevalence of immune-related differentially expressed genes and pathways in treatment-naïve LT tumor transcriptomic profiles. Expression of established immune compartment markers and an additional microglial signature were profiled in the SurVaxM cohort (Fig. 3A) [41, 42]. A subset of nGBM tumors primarily composed of mesenchymal subtype displayed global upregulation of numerous markers indicating a tumor microenvironment highly infiltrated by immune cells. However, this overall immune signature does not correspond with OS outcomes upon SurVaxM treatment. To study the contributions of individual immune compartments towards OS, TIMER2.0 immune deconvolution was applied to all samples [19, 20]. The signature scores for memory B cells (two-sided Wilcoxon rank sum test; *p* = 0.046), class-switched memory B cells (*p* = 0.013), non-regulatory CD4^+^ T cells (*p* = 0.029), and regulatory T cells (Tregs; *p* = 0.019) were significantly elevated in LT compared to ST (Fig. 3B). In contrast, ST tumors had significantly greater signature scores for common lymphoid progenitors (*p* = 0.013; Fig. 3B).

### Intratumoral B cells in nGBM associate with long-term survival upon SurVaxM treatment

**Fig. 4:**
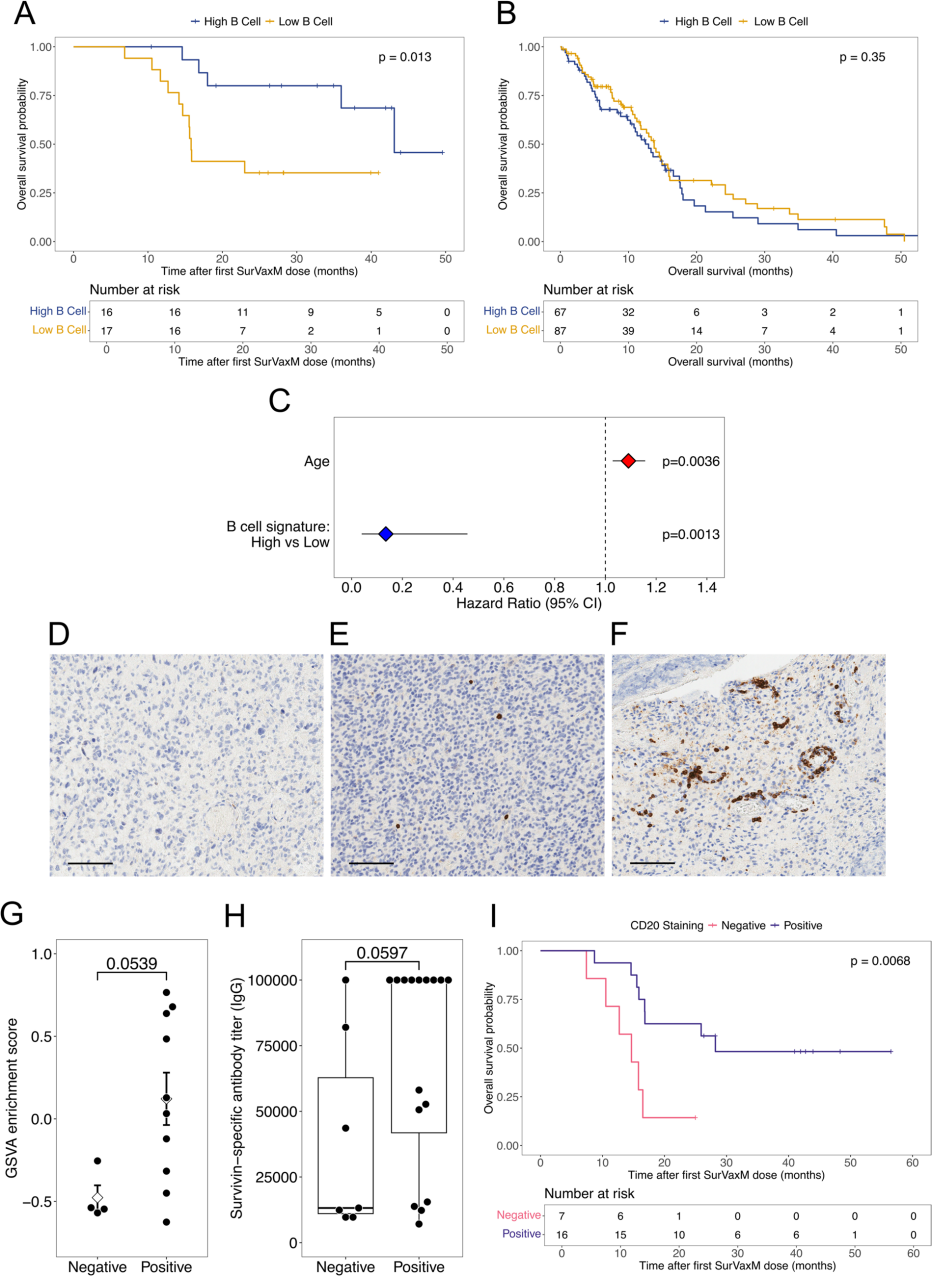
*Characterization of B cell compartment in SurVaxM treated patients* Kaplan Meier analysis of OS for the SurVaxM cohort (**A**; n = 33) or TCGA GBM dataset (**B**; n = 154) stratified by positive (high B cell signature; blue) or negative (low B cell signature; gold) gene set variation analysis score of 8-gene B cell expression signature. Respective p-values reported for log rank statistic. **C** A multivariate Cox proportional hazards model stratified by *MGMT* methylation status determined hazard ratios for age (HR = 1.091, 95% CI = 1.029–1.157) and B cell transcriptomic signature (high versus low, HR = 0.135, 95% CI = 0.0399–0.457) significantly associated with OS. Representative micrographs of CD20 IHC staining of FFPE GBM samples (n = 23) with negative (**D**, n = 7), rare positive (**E**, n = 7), or focal positive (**F**, n = 9) staining patterns. Images obtained at 15X magnification and scale bar represents 100 µm. **G** Comparison of modified B cell signature GSVA score across negative and positive (rare and focal positive) CD20 IHC staining patterns for all samples with transcriptomic data (n = 14, mean ± standard error of the mean, Wilcoxon rank test). **H** Maximum achieved survivin-specific antibody titer as measured by enzyme-linked immunosorbent assay of patient serum samples post-vaccination are compared across negative and positive categories of CD20 IHC staining (n = 23, Wilcoxon rank test) [8]. Boxplots represent the median and interquartile range. **I** Kaplan Meier analysis of OS stratified by negative (pink) or positive (purple) CD20 IHC staining in GBM tissue samples (n = 23, log rank test)

Three key pieces of data from expression profiling consistently support a greater presence of tumor associated B cells in SurVaxM LT survivors: (i) significant upregulation of *C4BPA*, an activating ligand of CD40 on antigen presenting cells; (ii) significant enrichment of humoral and complement related pathways by GSEA; and (iii) significantly higher immune deconvolution scores for memory and class-switched memory B cells. Furthermore, a growing body of evidence describes a positive correlation between B cell signatures and favorable clinical outcomes for a multitude of cancer types treated with immune checkpoint blockade (ICB); however, this association has not been similarly investigated in the context of tumor antigen targeted vaccines and GBM [43–46]. A modified B cell signature derived from Danaher et al. (2017) [41] and Budczies et al. (2021) [43] was assessed in the SurVaxM cohort expression profiles by GSVA and clustering (Fig. 3C). The collection of B cell markers consistently clustered more positively with LT than with ST survivors. High relative expression of the B cell signature in treatment-naïve tumors predicts long-term OS with SurVaxM treatment (Kaplan–Meier log rank test, *p* = 0.013); however, the same B cell signature is not predictive of OS in the TCGA cohort that only received standard of care without any form of immunotherapy (Kaplan–Meier log rank test, *p* = 0.35; Fig. 4A and B). In assessing the predictive potential of the intratumoral B cell expression signature, the covariates age, *MGMT* methylation status, and B cell signature score were determined to be significantly associated with OS by univariate Cox regression analysis, whereas, sex, *IDH1* mutational status, and molecular subtype were not significant in univariate Cox regression analyses of OS (Supplementary Table 2). An elevated B cell signature expression score positively associates with OS in a multivariate Cox proportional hazards model accounting for age as a covariate and stratified on *MGMT* methylation status (HR = 0.135, 95% CI = 0.0399–0.457, *p* = 0.00128; Fig. 4C).

To validate B cell transcriptomic data at a cellular level, a subset of tumor samples with available archived tissue (LT = 7 and ST = 7) were immunohistochemically stained for the B cell marker, CD20, resulting in identification of three distinct staining patterns. Samples were entirely negative for CD20 (Fig. 4D), displayed rare positive staining (Fig. 4E), or contained focal positive regions of CD20 staining (Fig. 4F) [47]. Focal CD20 staining of GBM tissue commonly, but not exclusively, present as organized foci in perivascular spaces. A transcriptomic signature for endothelial cells from the Human Protein Atlas (*SELE*, *SLC2A1*, *CD34*, *PECAM1*, and *VWF*) positively correlates with the B cell expression signature; although, high expression of the endothelial signature is not associated with improved OS (Supplementary Fig. 3C and 3D). Samples with a positive B cell transcriptomic signature also tended to score positively for CD20 staining, either rare or focal positive patterns (Fig. 4G). An additional 9 samples were assessed for CD20 IHC from patients with available tissue who were also enrolled in the SurVaxM trial but did not have associated molecular profiling data for their tumors. The most recent analysis of phase IIa SurVaxM clinical data identified a significant positive correlation between vaccine induced production of anti-survivin antibody and progression free survival (PFS) [8]. Notably, CD20 IHC staining for intratumoral B cells is concordant with the maximally obtained anti-survivin antibody titre of patients post-vaccination (Fig. 4H). Finally, the presence of intratumoral B cells identified by CD20 IHC in GBM tumors, prior to treatment, stratified patients for long-term OS (Kaplan–Meier log rank test, *p* = 0.0068, Fig. 4I).

## Discussion

**Fig. 5:**
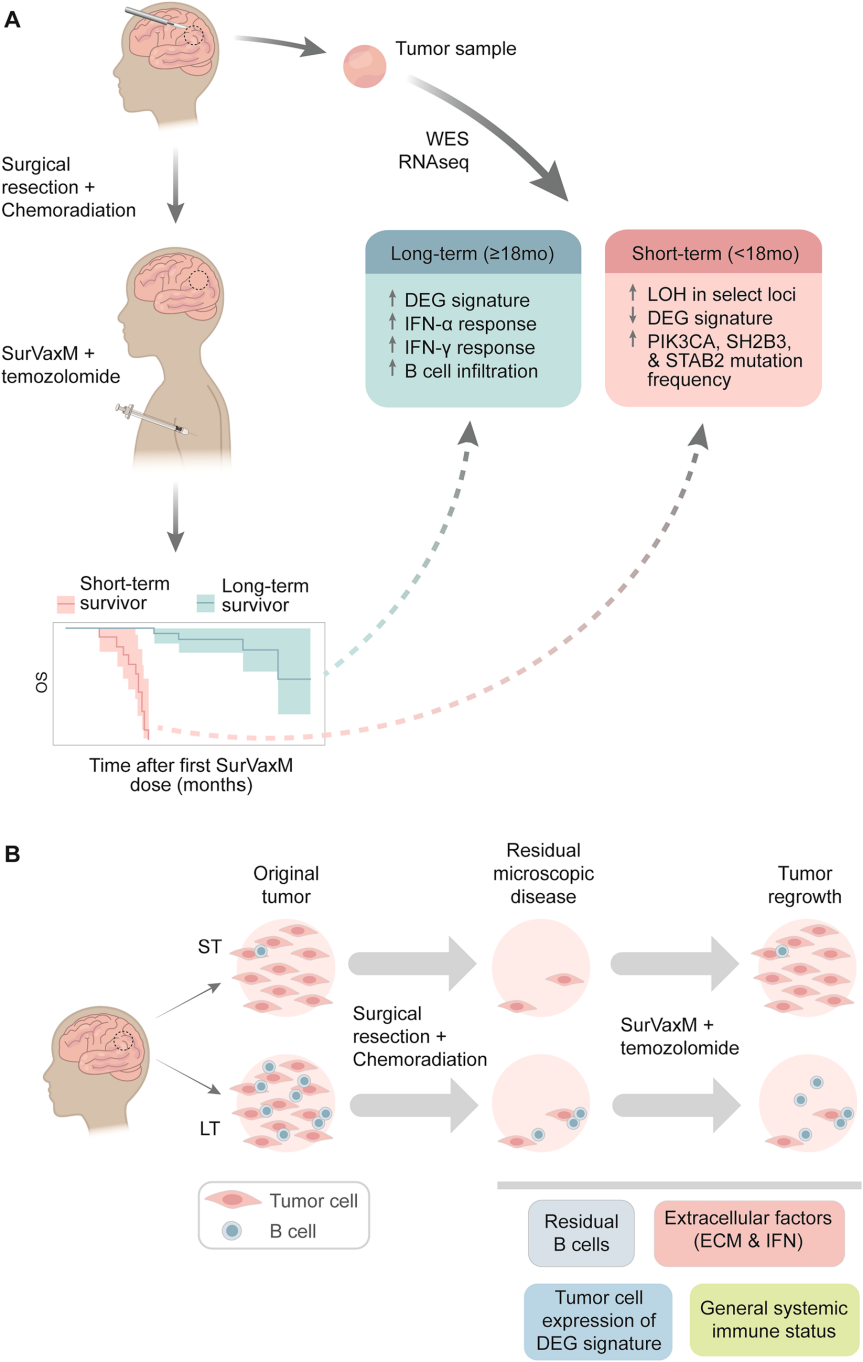
*Schematic overview of the study* **A** Retrospective genomic and transcriptomic analyses of GBM tumors from patients treated with SurVaxM plus temozolomide identified features associated with survival time. Tumors from patients surviving longer showed increased expression of interferon response, humoral response, extracellular matrix and glycan related gene signatures, along with increased infiltration of B cells. **B** Theoretical model for mechanism of factors influencing SurVaxM outcomes. Greater survival times in LT patients imply slower regrowth of the tumor from residual microscopic disease post-surgery. Retention of some of the cell and molecular differences seen in the pretreatment tumor (i.e. enhanced B cell residence and/or tumor cells expressing the DEG signature) in residual tissue may serve to diminish tumor regrowth in LT patients. Interplay between various factors, including those identified in this study, likely contribute to the diminished tumor regrowth in LT patients

The peptide vaccine SurVaxM has shown promise as a therapeutic agent in nGBM; 63 patients treated with SurVaxM plus TMZ showed mPFS of 11.4 months and mOS of 25.9 months in a recent single-arm clinical trial [8]. Within a discovery cohort representing approximately half of patients enrolled in the initial trial, genetic and transcriptomic tumor features were compared between patients who survived longer (OS ≥ 18mo) or shorter (OS < 18mo) times to identify potential predictors of response when treated with standard of care plus SurVaxM (Fig. 5A).

While it is recognized that the findings reported herein are limited by sample size and availability, the cohort was representative of the broader clinical trial cohort with comparable representation of sexes, median age, median survival times, and molecular subtypes; including *MGMT* methylation status, *IDH1* mutation, and *IDH1* wild type expression based subtypes [8]. Well-established GBM-specific molecular features were identified across all samples, including frequent mutations in *EGFR*, *TP53*, and *PTEN* genes; copy number gains of *EGFR*; and copy number loss of *PTEN*, *CDKN2A*, *CDKN2B*, and *MTAP* [3, 4, 24]. In addition, strong expression signature-based clustering of individual tumors identified IDH-mutant, classical, proneural and mesenchymal subtypes represented in expected proportions [4, 5]. In line with previous studies, these molecular subtypes did not associate with OS in patients treated with SurVaxM plus TMZ [5, 48]. Age and *MGMT* methylation correlated significantly with OS within our cohort. While it is well-established that patients with methylated *MGMT* tumors survive longer when treated with TMZ, SurVaxM treatment was shown to provide a survival benefit for both methylated and unmethylated *MGMT* patients [8].

In expression analysis, nine genes were found to be significantly upregulated in tumors of LT survivors. Five of these—*TBC1D3F*, *UMODL1*, *FUT2*, *MFAP4*, and *ALX4*—form a predictive signature for OS across patients treated in this study. Importantly, the signature was not found to be predictive of OS in GBM tumors within the TCGA database, indicating its potential specificity for treatment with SurVaxM. Many of the differentially expressed genes upregulated in LT survivors have been implicated as immune modulating and tumor-suppressive genes in gliomas and other cancer types. For example, aristaless-like homeobox 4 (*ALX4*) is a pro-apoptotic tumor suppressor that negatively regulates proliferation, colony formation, epithelial-to-mesenchymal transition (EMT), and invasion in hepatocellular, colorectal, lung, and breast carcinomas [49–53]. Other LT upregulated genes indicate substantial tissue remodeling in the tumor microenvironment by microfibril associated proteins (*MFAP4*) and fucosylation (*FUT2*) of key extracellular proteins, including selectins, that drive immune infiltration, antigen processing/presentation, and macrophage polarization [33–37, 54]. Further mechanistic studies are required to dissect the contributions of these genes towards SurVaxM response and long-term survival outcomes in GBM patients.

Comparisons of tumor immunological landscapes between survival groups revealed key differences associated with better survival outcomes following SurVaxM-TMZ treatment. In general, the immune composition of GBM includes many myeloid-derived cells such as microglia and macrophages, but few lymphocytes [55]. Significant enrichment of interferon gamma and interferon alpha response signatures were seen in tumors of LT survivors, indicating greater overall immunological activity. LT tumors also showed missense and gain of stop mutations in the *CSMD3* complement control gene, the loss of which is associated with enhanced complement protein expression in the brain [31].

Immune deconvolution analysis and a focused assessment of B cell gene set enrichment revealed significantly greater B cell expression signatures in LT survivor tumors compared with ST survivor tumors while accounting for age and *MGMT* methylation status. Along with significant enrichment of B cell transcriptomic signatures, CD20 IHC staining of tumors further established a positive association between B cell infiltration and OS. CD20 staining was limited to 23 tissue samples; however, 16/23 (70%) of these GBM tumors stained positive for CD20 indicating the presence of intratumoral B cells in proportions similar to those seen in a larger analysis of CD20 staining in GBM samples undertaken by others [56].

B cell infiltration is associated with improved survival across many tumor types, including melanoma, breast, colorectal, gastric, lung, and hepatocellular carcinomas [57]. In the context of adoptive cell transfer immunotherapy, B cells have been shown to cooperate with CD4^+^ T helper cells and dendritic cells to achieve durable anti-tumor responses through cytokine production and antigen presentation, respectively [58, 59]. B cells are known to have an immune modulatory function in the central nervous system and as key drivers of neuroinflammatory diseases [60]. Furthermore, mouse glioma models have demonstrated both pro- and anti-tumor functions for B cells. In the GL261 orthotopic GBM mouse model, B cells isolated from tumors were found to prevent CD8 + T cell proliferation in vitro [56]. However, another study found B cells to be essential activators of T cells against gliomas in mice, specifically functioning as antigen presenting cells (APCs) [61]. SurVaxM is known to stimulate humoral responses in addition to CD8^+^ and CD4^+^ T cell activation, and anti-SurVaxM antibody production correlated with OS, implying that B cell stimulation is likely to be an important element of SurVaxM’s mechanism of action [8, 62, 63]. The mechanistic link between greater intratumoral B cell infiltration and responsiveness to systemic SurVaxM immunotherapy is presently unclear, however one possible explanation is that B cells are key initiators and integral components of tumor-associated tertiary lymphoid structures (TLS). TLS consist of perivascular immune cell aggregates enriched in B cells and are associated with better prognoses and responsiveness to immunotherapies [64]. TLS have been found in both patient gliomas and in glioma-bearing mice where their formation was found to be enhanced by injection of agonistic α-CD40 antibody [65]. Interestingly, TLS have also been observed in SurVaxM treated GBM patients [63]. Notably, tumors from LT survivors expressed high levels of C4BP, a key ligand of CD40 and previously shown to increase leukocyte infiltration, promote B cell class switching, induce germinal center formation, and drive expansion of multiple antitumor immune compartments [38–40]. The presence of B cell-enriched aggregates within pre-treatment tumors may be indicative of an underlying propensity for TLS formation that supports enhanced APC priming and T cell activation.

The results described here suggest that nGBM tumors express molecular features which may be useful for patient stratification or prognostic prediction in the context of SurVaxM treatment and possibly other immunotherapies in the future. Overall, our studies suggest that sensitivity of nGBM to immunotherapy may be determined by the pre-existing immune status of the tumor, specifically the presence of interferon responsive genes and the presence of B cells (Fig. 5B). Additional studies are presently underway to assess the exomic and transcriptomic characteristics of tumors and resultant response to SurVaxM in a larger, randomized-controlled trial to validate and refine the findings reported here.

## Supplementary Information

Below is the link to the electronic supplementary material.

Supplementary file1 (PDF 1171 KB)

Supplementary file2 (CSV 3078 KB)

Supplementary file3 (DOCX 18 KB)

## Data Availability

Under New York State law, sequencing read files are not available to ensure confidentiality and protect the privacy of participants that did not consent to broad sharing of potentially identifiable data. Individuals interested in accessing summary level or raw sequencing data for replication or further analysis may request access through the corresponding author and the Institutional Review Board (IRB) of Roswell Park Comprehensive Center, subject to approval and compliance with ethical guidelines. The authors confirm that all other data supporting the findings of this study are available within the article and its supplementary materials. Open-access tier data generated by TCGA are publicly available from https:/portal.gdc.cancer.gov.

## Abbreviations

APC: Antigen Presenting Cell
CI: Confidence Interval
CNA: Copy Number Alterations
ECM: Extracellular Matrix
FFPE: Formalin Fixed and Paraffin Embedded
GBM: Glioblastoma
GDC: Genomic Data Commons
GSEA: Gene Set Enrichment Analysis
GSVA: Gene Set Variation Analysis
IHC: Immunohistochemistry
LT: Long-term Survivor
mOS: Median Overall Survival
mPFS: Median Progression Free Survival
MSigDB: Molecular Signatures Database
nGBM: Newly Diagnosed Glioblastoma
OS: Overall Survival
PFS: Progression Free Survival
ST: Short-term Survivor
TCGA: The Cancer Genome Atlas
TLS: Tertiary Lymphoid Structure
TMZ: Temozolomide
TPM: Transcripts per Million
