# Supplementary material for: Intratumoral B cell and interferon signatures in newly diagnosed glioblastoma are associated with longer survival in patients treated with SurVaxM
Source: Cancer Immunol Immunother. 2025 Oct 9;74(11):332. doi: 10.1007/s00262-025-04193-y (PMC12511503; doi:10.1007/s00262-025-04193-y)

Supplementary Figure 1.

(**A**) Kaplan Meier analysis of OS for the SurVaxM treated cohort stratified as LT (OS≥18 mo; blue) and ST (OS<18 mo; red) survivors. Significant p-value reported from the log rank test (n=33, p<0.0001). (**B**) Kaplan Meier analysis of OS for the SurVaxM treated cohort stratified by molecular subtype[5] as determined by gene set variation analysis. A non-significant p-value is reported from the log rank test (n=33, p=0.45).

Supplementary Figure 2.

(**A**) Occurrence of CNAs for individual genes across all chromosomes as a percentage of each OS class (blue=LT; red=ST) in a genome-wide stacked bar plot. Commonly altered genes in GBM are highlighted in black text and gene CNAs enriched in at least 3 LT or ST samples are highlighted in gold text. (**B**) Oncoprint of copy number alterations for genes altered in at least 3 samples and uniquely enriched in either LT or ST tumors (HETLOSS=heterozygous loss; HOMDEL=homozygous deletion). Rows are grouped by indicated chromosome locations for each gene. Annotations denote OS class, OS in months from initial SurVaxM dose, assigned molecular subtype[5], and methylation status of *MGMT*. Row-wise frequency of gene alterations are indicated by percentages and vertical bar plot.

Supplementary Figure 3.

(**A**) Principal components plot of PC1 (18.73% of variation) and PC2 (12.87% of variation) for all RNA-seq analyzed tumor samples (n=33) with molecular subtype[5] indicated by color (classical=red, mesenchymal=blue, proneural=green, or IDH1 mutant=orange). (**B**) Boxplots of DESeq2 normalized expression counts and statistics (LFC=$\log_2$ fold change; adj.p=FDR adjusted

p-value) for all differentially expressed genes between LT (n=20, blue) and ST (n=13, red) OS classes. Boxplots represent median and interquartile range. (**C**) Correlation plot of B cell and endothelial transcriptomic GSVA signature scores for molecularly profiled GBM samples colored by OS class (n=33; ST=red, blue=LT, grey=95% CI; Pearson correlation). (**D**) Kaplan Meier analysis of molecularly profiled GBM samples stratified by high (positive GSVA score; red) or low (negative GSVA score; green) endothelial expression signature (n=33, log rank test).

Supplementary Figure 1

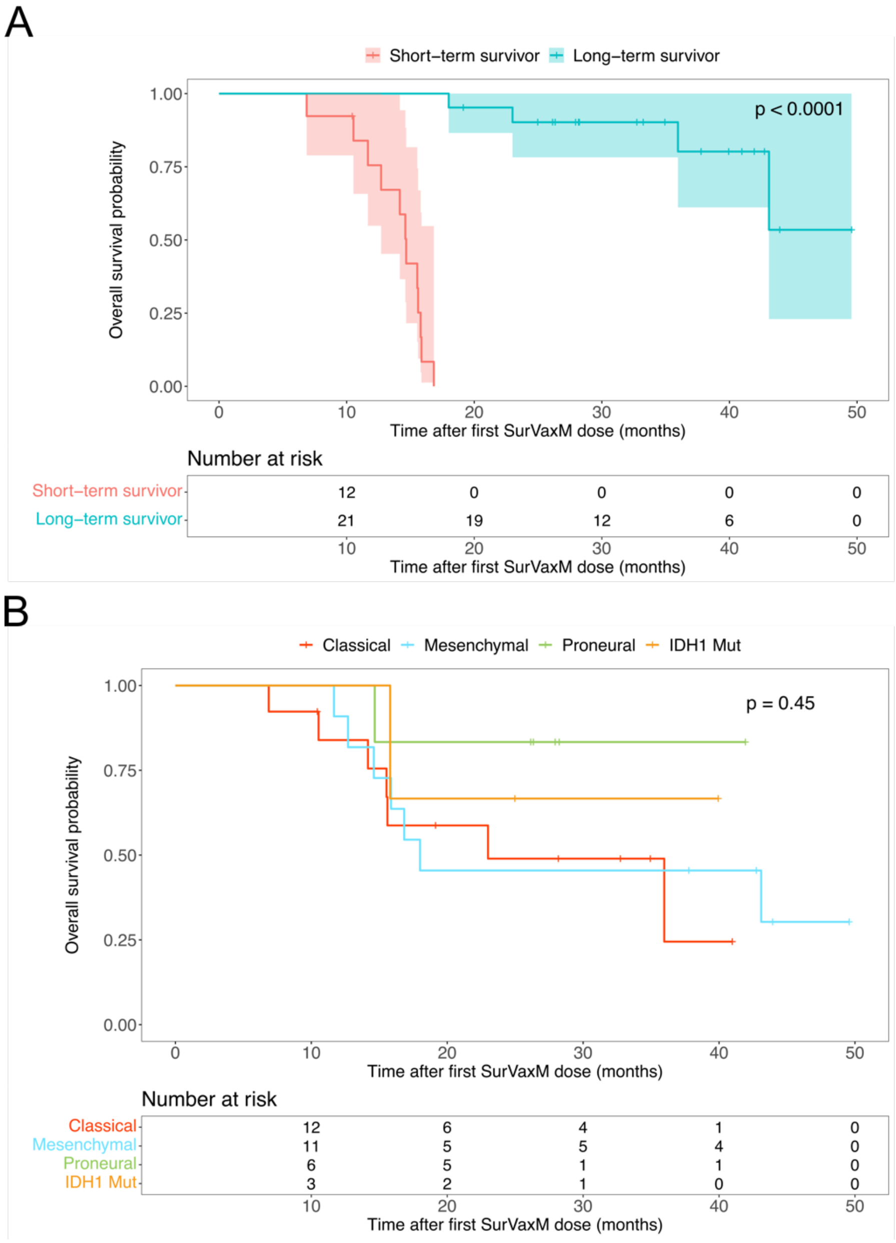

Supplementary Figure 2

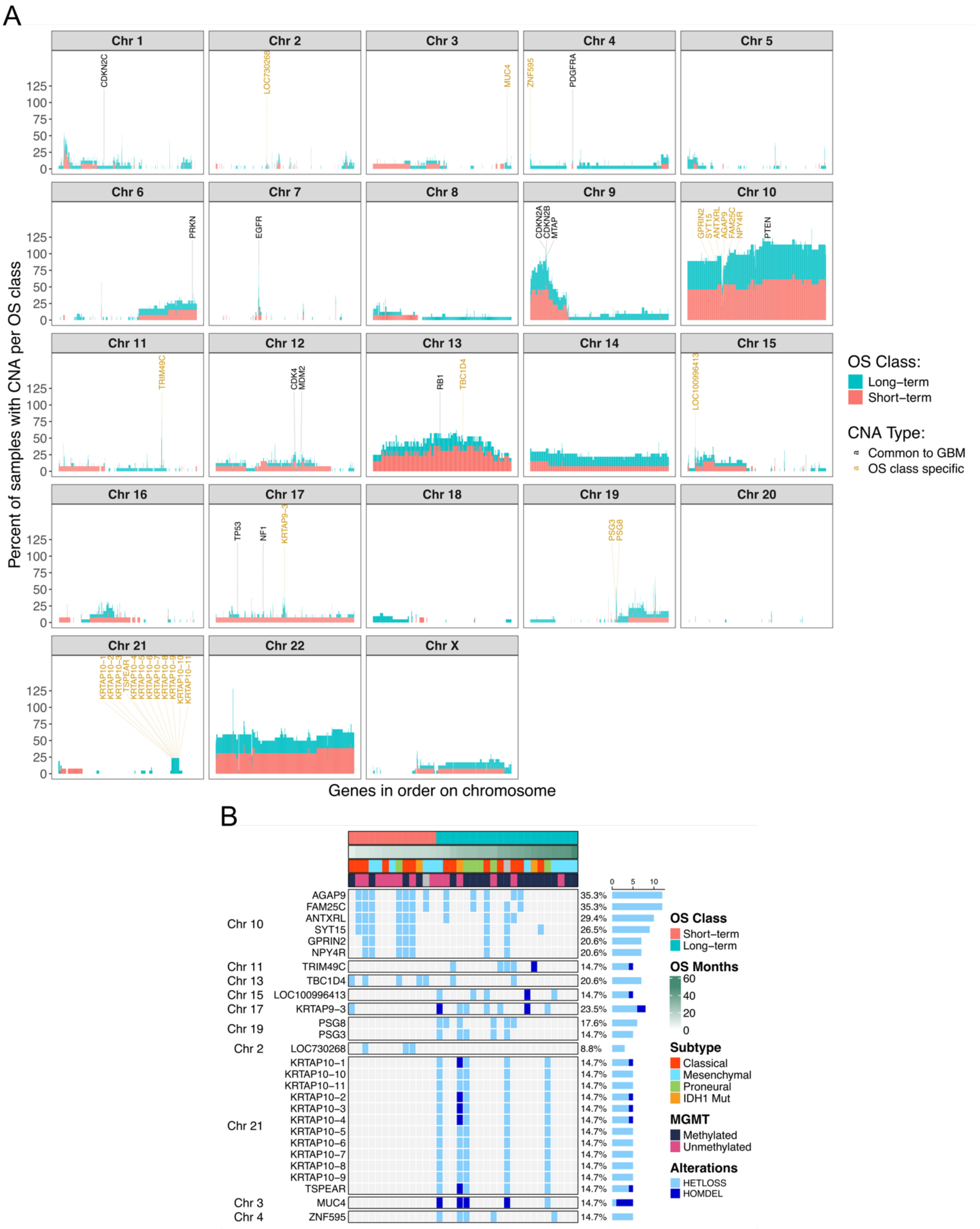

Supplementary Figure 3

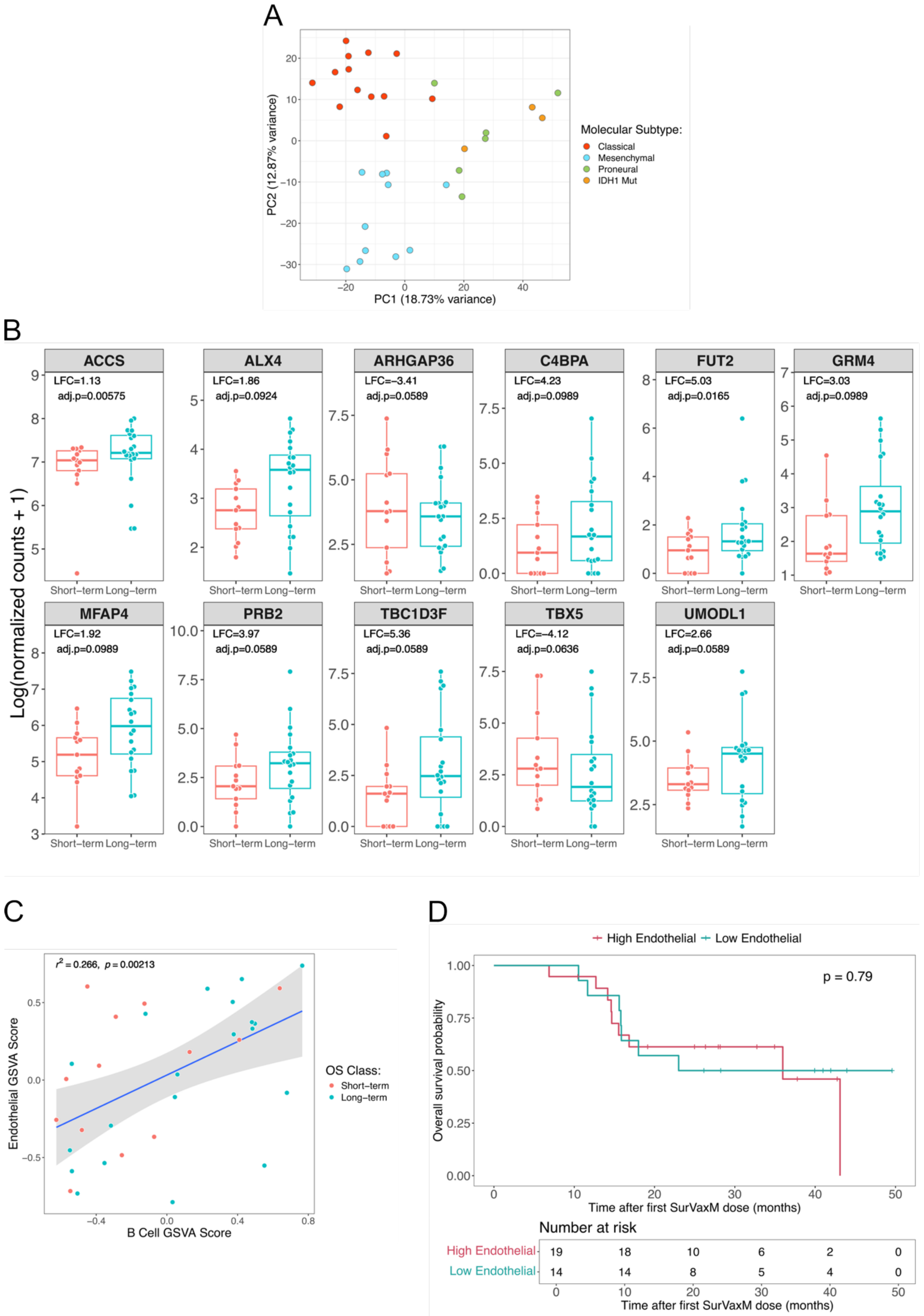

Supplement: Supplementary file 1 — Supplementary file1 (PDF 1171 KB) [file 262_2025_4193_MOESM1_ESM.pdf]
