# Supplementary material for: Intratumoral B cell and interferon signatures in newly diagnosed glioblastoma are associated with longer survival in patients treated with SurVaxM
Source: Cancer Immunol Immunother. 2025 Oct 9;74(11):332. doi: 10.1007/s00262-025-04193-y (PMC12511503; doi:10.1007/s00262-025-04193-y)
Supplement: Supplementary file 3 — Supplementary file3 (DOCX 18 KB) [file 262_2025_4193_MOESM3_ESM.docx]

**Supplementary Table 2.** Univariate Cox regression for OS.

|  | N | HR^1^ | 95% CI^1^ | p-value |
| --- | --- | --- | --- | --- |
| **Age** | 33 | 1.07 | 1.01, 1.14 | 0.018 |
| **Sex** | 33 |  |  |  |
| Female |  | — | — |  |
| Male |  | 0.60 | 0.22, 1.67 | 0.3 |
| **MGMT Status** | 32 |  |  |  |
| Methylated |  | — | — |  |
| Unmethylated |  | 2.99 | 1.04, 8.60 | 0.043 |
| **Molecular Subtype** | 33 |  |  |  |
| Classical |  | — | — |  |
| Mesenchymal |  | 0.83 | 0.28, 2.49 | 0.7 |
| Proneural |  | 0.22 | 0.03, 1.83 | 0.2 |
| IDH1 Mut |  | 0.45 | 0.05, 3.63 | 0.4 |
| **B Cell Signature** | 33 |  |  |  |
| Low B Cell |  | — | — |  |
| High B Cell |  | 0.25 | 0.08, 0.81 | 0.020 |
| **IDH1** | 33 |  |  |  |
| Wild Type |  | — | — |  |
| Mutant |  | 0.59 | 0.08, 4.52 | 0.6 |
| ^1^HR = Hazard Ratio, CI = Confidence Interval | | | | |
